# Robotic Surgery in Severely Obese Frail Patients for the Treatment of Atypical Endometrial Hyperplasia and Endometrial Cancer: A Propensity-Match Analysis at an ESGO-Accredited Center

**DOI:** 10.3390/cancers17030482

**Published:** 2025-02-01

**Authors:** Martina Arcieri, Federico Paparcura, Cristina Giorgiutti, Cristina Taliento, Giorgio Bogani, Lorenza Driul, Pantaleo Greco, Alfredo Ercoli, Vito Chiantera, Francesco Fanfani, Anna Fagotti, Giovanni Scambia, Andrea Mariani, Stefano Restaino, Giuseppe Vizzielli

**Affiliations:** 1 Clinic of Obstetrics and Gynecology, “Santa Maria della Misericordia” University Hospital Azienda Sanitaria Universitaria Friuli Centrale, 33100 Udine, Italy; martina.arcieri@asufc.sanita.fvg.it (M.A.); giuseppe.vizzielli@uniud.it (G.V.); 2 Department of Medicine (DMED), University of Udine, 33100 Udine, Italy; 3 Obstetrics and Gynecology Unit, Department of Medical Sciences, University of Ferrara, 44121 Ferrara, Italy; 4 Department of Development and Regeneration, Woman and Child, KU Leuven, 3000 Leuven, Belgium; 5 Gynaecological Oncology Unit, Fondazione IRCCS Istituto Nazionale dei Tumori di Milano, 20133 Milan, Italy; 6 Department of Human Pathology in Adult and Childhood “G. Barresi”, University of Messina, 98122 Messina, Italy; 7 Unit of Gynecologic Oncology, National Cancer Institute, IRCCS, Fondazione “G. Pascale”, 80131 Naples, Italy; 8 Gynecologic Oncology Unit, Department of Woman and Child Health and Public Health, Fondazione Policlinico Universitario Agostino Gemelli IRCCS, 00136 Rome, Italygiovanni.scambia@policlinicogemelli.it (G.S.); 9 Department of Obstetrics and Gynecology, Mayo Clinic, Rochester, MN 55905, USA; 10 PhD School in Biomedical Sciences, Gender Medicine, Child and Women Health, University of Sassari, 07100 Sassari, Italy

**Keywords:** endometrial cancer, atypical endometrial hyperplasia, robotic surgery, laparoscopic surgery

## Abstract

Background and Objectives: Robotic-assisted laparoscopy (RS) has been widely adopted for the management of endometrial cancer (EC) due to favorable perioperative outcomes, especially in the context of obesity, which is an established risk factor for EC. This study retrospectively evaluated the perioperative outcomes of RS versus conventional laparoscopy (LS) in treating EC and atypical endometrial hyperplasia (AH). Methods: Between November 2021 and October 2023, 138 patients with AH or EC underwent surgery at the Clinic of Obstetric and Gynecological—Azienda Sanitaria Universitaria Friuli Centrale, Udine. All patients had total hysterectomy with bilateral salpingo-oophorectomy, with or without lymphadenectomy or sentinel lymph node biopsy. The study included 62 patients treated with LS and 62 with RS. Results: The median BMI was higher in the RS group (35.5 vs. 24 kg/m^2^, *p* = 0.001). There was no significant difference in operative time between the laparoscopic group and the robotic console time (median 130 vs. 130 min, *p* = 0.131). No significant differences were found in terms of blood loss, conversion to laparotomy, intraoperative complications, hospital stay, or early postoperative complications between the two groups. Conclusions: Our data confirm the feasibility of robotic surgery in obese patients, allowing surgical results comparable to those of laparoscopy in normal-weight patients.

## Simple Summary

In recent years, robotic-assisted laparoscopy has become an important surgical approach for the management of endometrial cancer, particularly in obese patients. After a propensity-matched analysis, this study retrospectively compared the outcomes of robotic-assisted laparoscopy and conventional laparoscopy in the surgical staging of 62 patients in each group with endometrial cancer or atypical endometrial hyperplasia. Patients underwent total hysterectomy with bilateral salpingo-oophorectomy, with lymphnode staging. In our series, patients in the robotic group had a significantly higher median body mass index (35.5 vs. 24 kg/m^2^). Despite this, no significant differences were observed between the two groups in intra and postoperative outcomes. These results support the use of robotic-assisted laparoscopy as a feasible and effective surgical option, especially for obese patients.

## 1. Introduction

Endometrial cancer (EC) represents one of the most frequent gynecological malignancies worldwide, with 417,367 new cases and 97,370 deaths reported in 2020 [1]. EC is the only gynecological malignancy with increasing incidence and mortality, which is partly attributed to the rising prevalence of obesity and type 2 diabetes [2].

EC is frequently diagnosed when the disease is still limited to the uterus. For this reason, in the early stage of EC, the 5-year survival rate reaches approximately 90%, and surgical treatment is essential for the cancer’s comprehensive staging and potentially represents the principal curative management [3].

The standard treatment involves a primary hysterectomy with bilateral salpingo-oophorectomy. Lymph node staging depends on histological factors, the disease stage, and patient characteristics [4]. The main guidelines [5,6,7] recommend minimally invasive surgery (MIS) when technically feasible [5,6,7]. Several studies suggest that laparoscopic surgery (LS) offers advantages over laparotomy, including less blood loss, a lower complication rate, faster postoperative recovery, and a shorter hospital stay without compromising oncological outcomes in EC [5,6,7].

Since its approval in 2004, robotic-assisted laparoscopy (RS) has been widely adopted in gynecologic surgery, bringing numerous benefits due to 3D vision, increased precision and skill, improved ergonomics, and reduced tremors [8]. In addition, the learning curve associated with robotic surgery is shorter [9,10]. The overall feasibility of robotic-assisted laparoscopy for the management of EC has been amply demonstrated. This approach has favorable perioperative outcomes compared to conventional laparoscopy and laparotomy. However, despite extensive studies, the results in the literature remain controversial [8,11,12,13,14]. RS has the potential to enable more patients to benefit from the minimally invasive approach, such as those at high risk of anesthetic complications, including those suffering from obesity, patients of advanced age+, and those with medical comorbidities [15]. RS may be a helpful tool in the context of obesity, which is a well-established risk factor for EC [16,17,18]. The improved dexterity of the robotic arms compared to standard laparoscopy is very important in reducing the technical challenges of complex surgical steps.

The European Society of Gynecological Oncology (ESGO) accreditation for centers specializing in EC surgery recognizes institutions that possess the necessary skills, experience, organizational capacity, and commitment to providing the highest standards of surgical care for women to optimize and ensure the quality of care and to improve the management and outcome of patients with EC. Elements evaluated include the percentage of patients with early EC treated with MIS and those with a BMI > 35 kg/m^2^ undergoing MIS. Starting from these premises, we conceptualized a retrospective single-center study to evaluate and track the potential differences between robotic-assisted laparoscopy and the laparoscopic approach in surgery for EC and atypical endometrial hyperplasia in terms of perioperative outcomes.

The primary endpoint of our study was to compare the intraoperative outcomes of RS versus LS. As secondary endpoints, we evaluated differences in postoperative outcomes and complications within 30 days of the procedure.

## 2. Materials and Methods

### 2.1. Patients

Our institutional review board approved this monocentric retrospective cohort study (IRB: 44/2024 of 7 February 2024). We analyzed patients diagnosed with atypical endometrial hyperplasia or endometrial cancer undergoing robotic and laparoscopic surgery between November 2021 and October 2023 at the Clinic of Obstetric and Gynecological—Azienda Sanitaria Universitaria Friuli Centrale, Udine (Italy), an ESGO-accredited center for the surgical treatment of EC.

All patients provided written consent for the use of their data for research purposes, and our institutional review board approved this monocentric retrospective study. All procedures involving human participants were conducted in accordance with the ethical standards of the institutional and national research committee and in compliance with the 1964 Helsinki Declaration and its later amendments or comparable ethical standards.

The inclusion criteria for this study were a diagnosis of atypical endometrial hyperplasia or early endometrial cancer confirmed by definitive histological examination and surgery performed using a minimally invasive approach (laparoscopic or robotic). The exclusion criteria were patients with advanced EC who underwent laparotomic cytoreduction surgery and lack of informed consent to participate in the study.

All patients underwent pelvic ultrasonography before surgery, and we took blood samples according to the internal protocol (blood count and renal and hepatic function). Women with EC also underwent a preoperative thoracoabdominal CT scan.

For every patient, the following information was gathered: age at surgery, body mass index (BMI), FIGO staging (2023), molecular characterization, lymph node assessment (i.e., systematic lymphadenectomy or sentinel lymph node technique (SLN), operative time (OT), docking time and console time in robotic surgery, estimated blood loss, postoperative intensive care unit admission, postoperative hospital stay, hospital readmission, and intraoperative and postoperative complications.) In our center, molecular characterization was obtained by hysteroscopy biopsy analysis, if feasible, as previously published [19].

Operative time (OT) was the interval between the skin incision and suture. Docking time (DT) was required to move the patient cart into the surgical field, set the four robotic arms on the related robotic trocars following the standardized configuration, and insert the robotic instruments in the abdomen. The console time was counted from the moment the first operator started the procedure at the robotic console until the end of its use.

According to Dindo’s classification, intraoperative and postoperative complications were defined as adverse events occurring during surgery or within the first four postoperative weeks [20].

No specific preoperative data (e.g., age, cancer stage at preoperative imaging, non-endometrioid histology, etc.) influenced the choice between LS and RS, except for patients with a BMI > 25 kg/m^2^, who were preferably selected for the robotic approach.

All women were submitted to total hysterectomy with bilateral salpingo-oophorectomy with or without systematic lymphadenectomy or sentinel lymph node technique (SLN), according to international guidelines [21,22].

In obese patients, to facilitate access to the abdominal cavity, surgeons performed a left subcostal access with an optical trocar.

Two expert gynecologic oncologists (GV and SR) who had undertaken a minimum of 200 cases of endometrial cancer surgery before this study performed all procedures.

The robotic platform used was The da Vinci^®^ Xi Surgical System (Intuitive Surgical, Sunnyvale, CA, USA).

### 2.2. Statistical Analysis

Due to the nonrandomized nature of the study design and potential allocation biases arising from the retrospective comparison between groups (RS vs. LS), we performed a propensity-matched analysis.

This method aims to estimate the treatment effect by accounting for factors (e.g., constitutional variables) that predict treatment assignment, thereby reducing biases from different covariates.

A propensity score was developed using a multivariable logistic regression model, including variables such as age and history of previous surgery. Patients undergoing RS were matched 1:1 with patients undergoing LS using a caliper width of less than 0.1 standard deviations of the estimated propensity score’s logit odds. The sample size after matching was 124 (62 cases and 62 controls controls). Detailed information on propensity matching can be found elsewhere [23]. Clinicopathological and demographic characteristics were described using descriptive statistics. Qualitative variables were presented as frequencies and percentages, while quantitative variables were summarized as means and medians. The χ2 analysis or Fisher’s exact test was used for categorical variables, and the Student’s t-test and Mann–Whitney test were applied to continuous variables, as appropriate. Differences between groups were considered statistically significant at *p* < 0.05 (95% confidence interval). The NCSS statistical software program, version 11.0 (NCSS Statistical Software, Kaysville, UT, USA), was used for the analysis.

## 3. Results

One hundred and forty-seven consecutive patients with atypical endometrial hyperplasia or endometrial cancer underwent surgery at the Clinic of Obstetrics and Gynecology, Azienda Sanitaria Universitaria Friuli Centrale of Udine, Italy, between November 2021 and October 2023.

**Figure 1:**
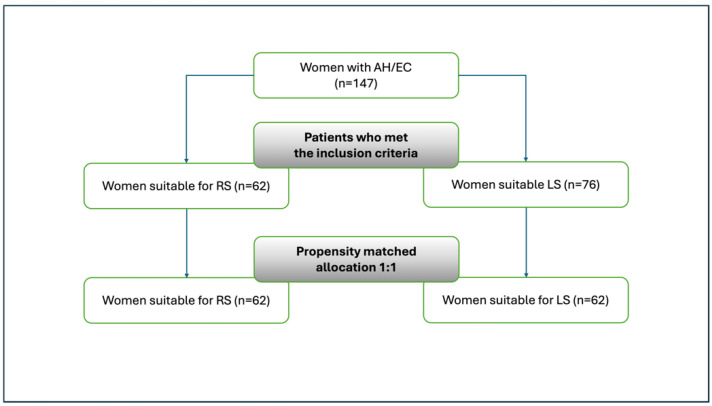
Study design and selection process. AH: atypical hyperplasia; EC: endometrial cancer; LS: laparoscopic surgery; and RS: robotic surgery.

Ten women were excluded because they were submitted to laparotomic cytoreduction surgery for advanced endometrial cancer. Out of 138 patients enrolled, 76 (55%) were treated by LS and 62 (45%) by RS (Figure 1). After propensity-match analysis, we evaluated 62 cases in both groups. 

Table 1 summarizes patients’ demographic, intraoperative, pathological, and oncological characteristics according to the type of surgery.

The median BMI was higher in the RS than in the LS group (35.5 vs. 24 kg/m^2^, *p* = 0.000).

Of 124 enrolled patients, 12.9% in the LS group and 19.4% in the RS group (*p* = 0.329) had atypical endometrial hyperplasia. No conversion to laparotomy (in the LS and RS groups) or to laparoscopy (in the RS group) was needed. No intraoperative complications occurred. The mean EBL was 68.5 mL in the RS group and 45.2 mL in LS (*p* = 0.136).

The lymph nodal assessment was performed more often in the LS group (82.3% vs. 66.1%; *p* = 0.04). In the LS group, it was obtained bilaterally in 49 cases, while in the RS group, it was obtained bilaterally in 38 patients (*p* = 0.031).

If we exclude atypical hyperplasia and endometrial cancer FIGO stage IA1 from the analysis, lymph nodal assessment was obtained in 48/53 (90.5%) and 29/43 (67.4%) patients (*p* = 0.005) (Table 2).

The study groups were significantly different in terms of the operative time. The laparoscopic procedures were shorter, with a median of 130 min (range = 77–240 min) compared with a median of 195 min (range, 120–310 min) for the RS procedures (*p* < 0.001). If we compare only the LS group’s operative time and the RS console time, no statistical difference was recorded (median 130 vs. 130 min, *p* = 0.131). The median docking time was 32 min (16–90 min).

Additional procedures were required for 27 patients in the LS group: 24 omental/peritoneal biopsies, 2 excisions of benign vulvar lesions, and 1 umbilical hernia repair. In the RS group, we recorded 8 additional procedures: 6 omental/peritoneal biopsies, 1 umbilical hernia repair, and 1 excision of benign vaginal and vulvar lesions.

Four patients in the RS group were transferred to the intensive care unit in the immediate postoperative period due to anesthesiologic issues.

There was no statistical difference between the two groups in terms of early postoperative complications (*p* = 0.052). In detail, we recorded seven early-surgery-related complications: in the LS group, one wound infection treated with an oral antibiotic and in the RS group, one postoperative stroke, two wound infections treated with an oral antibiotic, one urinary infection treated with an oral antibiotic, one vaginal cuff dehiscence requiring surgical repair, and one paresthesia of the left thigh treated with oral administration of nonsteroidal anti-inflammatory drugs.

The mean hospital stay was 2 and 3 days in the LS and RS groups, respectively (*p* < 0.001).

## 4. Discussion

MIS is considered the gold standard for treating EC and is characterized by reduced operative morbidity, shorter hospital stays, and a better quality of life [24,25,26]. Obesity is a well-recognized risk factor in the development of EC, but in these patients, MIS presents various challenges. These include technical issues related to gaining access to the abdominal cavity and achieving adequate exposure. In obese patients, it is crucial to account for the altered anatomical relationships. The weight of the panniculus frequently causes the umbilicus to shift downward, making traditional landmarks for port placement unreliable. To facilitate access to the abdominal cavity in obese patients, we performed a left subcostal access with an optical trocar. Factors such as pneumoperitoneum and the steep Trendelenburg position, necessary for surgical visualization, can negatively impact cardiopulmonary function and mechanical ventilation. Robotic-assisted surgery addresses these difficulties by providing an enhanced three-dimensional visualization, utilizing wristed instruments with seven degrees of motion to improve surgeon flexibility and precision, and optimizing operative technique and exposure. The fourth robotic arm allows greatly improved surgical field exposure in patients with significant visceral fat. Another reported advantage is the capacity of fixed mechanical arms to bear the weight of the abdomen, allowing robotic procedures to be conducted with lower insufflation pressures, thereby supporting prolonged ventilation during steep Trendelenburg positions [24,25].

Our data confirm the feasibility of robotic surgery in obese patients, allowing surgical results comparable to those of laparoscopy in normal-weight patients.

Based on several studies that demonstrated more significant advantages of RS over LS in obese patients, we generally assign RS to patients with BMI > 30 kg/m^2^ [27,28].

Recently, two retrospective studies demonstrated that robotic and laparoscopic surgery are superimposable in terms of oncological outcomes in the treatment of endometrial cancer [25].

Regarding operative time, RS took longer than LS in our cohort, confirming the assumptions in most of the literature [12,13,29,30]. However, the time difference between the two groups disappears when the time at the console is evaluated only as the “true” operating time. In other words, our results would make it seem that robotic surgery “normalizes” surgical times on fragile and obese patients, making it comparable to routine laparoscopic surgery. Moreover, we noted a significant decrease in docking time after the first 20 procedures performed [30]. Indeed, we must point out that while the first surgeons had a high level of experience in robotic surgery gained from previous experience in a center that has been using robotic surgery for more than ten years, the nurses and assistant surgeons had no experience in this field.

In our series, no cases required conversion to laparotomy. A recent review and meta-analysis [24] showed a conversion rate of about 6% in both laparoscopic and robotic surgery in obese patients with EC, mainly due to inadequate surgical field exposure and anesthesiologic complications. In our hospital, the two surgeons have extensive experience in robotic surgery, while the anesthesiology team dedicated to robotics has previous experience in bariatric surgery. Managing obese patients and the steep Trendelenburg position requires significant anesthesiologic expertise due to the intricate challenges and risks involved, particularly in terms of airway management and respiratory and cardiovascular complications.

This confirms the importance of a dedicated team, especially for complex cases.

Our data showed a significantly higher median length of hospital stay in the RS group, probably due to a higher rate of women being transferred postoperatively to the intensive care unit for anesthesiologic concerns and due to a postoperative stroke with an 11-day hospitalization period. Furthermore, it is known that the duration of hospitalization increases in obese patients. However, the median days of hospitalization were consistent with the data reported in the literature for both the RH and the LS group [28,31].

The rate of early postoperative complications was low, with no statistical differences between the two groups, in agreement with the literature. There was a slightly higher complication rate in the RS group, but these data are consistent with the higher median BMI [32]. Half of the complications in the RS group were infectious and may be related to metabolic syndrome. In our opinion, the slightly higher complication rate in the RS group is due to the type of patients (obese and very obese) and not to the surgical approach.

Our internal guidelines recommended performing SLN mapping in patients with atypical hyperplasia due to the high risk of diagnosing invasive EC on final histopathological analysis. However, lymphadenectomy is not performed if SLN mapping fails [33]. Excluding cases of atypical hyperplasia in preoperative biopsies and EC stage IA1 from the total number of patients without retroperitoneal staging occurred for only 4 cases in the LS group and 10 in the RS group. SLN or lymphadenectomy was mainly lacking in elderly or very obese patients. The literature reported that the SLN mapping rate was lower in obese patients [34]. Obesity has the potential to alter the migration of the tracer by impairing the normal function of the lymphatic vascular system, thereby compromising the effectiveness of the technique. A recent multicenter, propensity-matched, retrospective study highlighted a 1.156-fold increase in the risk of mapping failure for every five units of BMI increase (OR 1.156, 95% CI 1.033–1.294, and *p* = 0.012), resulting in a decrease in both bilateral mapping and the overall detection rate [35].

From an economic point of view, laparoscopic hysterectomy is the least expensive surgical option compared to robotic and open surgery. However, when considering societal costs linked with recovery time, robotic surgery proves to be less costly than abdominal hysterectomy. Moreover, robotic surgery costs decrease as the procedural volume rises [36]. In our hospital, the cost of robotic instrumentation is about 1890 euros per operation, while the price of each day of hospital stay is about 600 euros. According to a recent meta-analysis [37], robotic surgery reduces the length of hospital stay by approximately 3.5 days (compared to open surgery), reducing the cost of robotic surgery, decreasing complications, and allowing better patient recovery. A lower rate of conversion to laparotomy in the robotic group than in the laparoscopic group is reported in the literature, especially in very obese patients [28].

Robotic surgery also allows for the expanded use of MIS in obese patients, for whom the laparoscopic approach can be challenging (while open surgery is burdened with a higher incidence of complications and a longer length of stay), allowing for a reduction in the costs associated with hospitalization and complications. In addition, several robotic platforms have recently entered the market, resulting in competition and likely decreasing prices for robotic systems and disposables in the foreseeable future.

From the perspective of the need for a proper allocation of economic resources, currently laparoscopy, due to its lower costs, should be indicated for the EC treatment of patients with normal BMI, while for obese patients, due to the low laparotomy conversion rate, the most indicated surgical approach should be robotic surgery.

The limits of our study are the retrospective design and the initial “learning curve” of the operating room staff involved at the beginning of our experience. This may have impacted the operative times, especially the docking times.

The main strength was a team dedicated to robotic surgery in an ESGO-accredited center, which consequently allows for the standardization of procedures.

In conclusion, robotic surgery for EC appears to have benefits for specific patient populations, especially those with a high BMI, in whom laparotomic surgery is associated with higher morbidity, and where laparoscopic surgery might present several technical difficulties.

While waiting for an ongoing randomized trial comparing robotic and laparoscopic in obese patients with endometrial cancer [38], we suggest that RS is a valid option and might be considered an alternative to laparoscopic surgery in obese and frail women.

## 5. Conclusions

In conclusion, our results support the use of robotic-assisted laparoscopy in the treatment of endometrial cancer as a feasible and effective surgical option, especially for frail severely obese patients. Ongoing prospective randomized studies will be useful to confirm these data.

## Figures and Tables

**Table 1:** Demographic, intraoperative, pathological, and oncological characteristics according to the type of surgery.

	LS (*n* = 62)	RS (*n* = 62)	*p* Value
N° patients	N	N	
Median age, year (range)	64.5 (46–88)	66.5 (43–90)	0.269
Previous Abdominal Surgeries (%)	32 (51.6)	40 (64.5)	0.145
Median BMI, kg/m^2^ (range)	24 (18–33)	35.5 (23–67)	0.001
Atypical Hyperplasia	8 (12.9%)	12 (19.4%)	0.329
Endometrial cancer (FIGO 2023 stage)	54 (87.1%)	50 (80.6%)	
IA1	1	7	
IA2	29	25	
IA3	/	1	
IAm(POLE mut)	6	3	
IB	4	4	
IIA	2	1	
IIB	3	3	
IIC	5	2	
IICm(p53abn)	2	1	
IIIA1	/	1	
IIIA2	/	1	
IIIB2	1	0	
IIIC1ii	1	1	
Pelvic lymph node assessment			
No, N (%)	11 (17.7)	21 (33.9)	0.040
Yes, N (%)	51 (82.3)	41 (66.1)	
SLN			
unilateral, N (%)	7 (11.3)	5 (8.1)	0.543
bilateral, N (%)	37 (59.7)	31 (50)	0.279
Pelvic lymphadenectomy, N (%)			
unilateral, N (%)	5 (8.1)	2 (3.2)	0.243
bilateral, N (%)	7 (11.3)	5 (8.1)	0.543
Aortic lymphadenectomy, N (%)	2	0	0.154
Mean blood loss, ml (range)	45.2 (0–300)	68.5 (0–700)	0.136
Patients with intraoperative complications	0	0	
Conversion to laparotomy	0	0	
Conversion to laparoscopy	0	0	
Median OT, minutes (range)	130 (77–240)	195 (120–310)	<0.001
Median OT w/o console time, minutes (range)	130 (77–240)	130 (75–201)	0.131
ICU N (%)	0	4 (6.5)	0.042
Patients with early postoperative complications	1 (1.6)	6 (9.7)	0.052
Grade I	0	1 (16.7)	0.659
Grade II	1	3 (50)	0.350
Grade IIIa	0	0	/
Grade IIIb	0	1 (16.7)	0.659
Grade IV	0	1 (16.7)	0.659
Re-intervention	0	1 (1.6)	0.315
Median hospital stay, days (range)	2 (1–3)	3 (1–11)	<0.001
Re-admission	0	1 (1.6)	0.315

LS: laparoscopic surgery; RS: robotic surgery; BMI: body mass index; SLN: sentinel lymph node; OT: operative time; and ICU: intensive care unit.

**Table 2:** Patients without lymph node assessment.

Type of Surgery	Age	BMI	AH/FIGO 2023 Stage	Reason for Non-Lymphadnectomy
LS	81	25	IB	AH in preoperative biopsy
LS	59	22	AH	
LS	61	32	AH	
LS	83	20	IB	Old age and dementia
LS	51	21	AH	
LS	59	23	AH	
LS	63	26	AH	
LS	60	28	IIB	Myometrial infiltration of less than 50% on extemporaneous examination
LS	81	31	IB	BMI, old age, comorbidity
LS	84	27	IAm(POLE mut)	Synchronous metastatic breast cancer
LS	51	22	AH	
RS	58	54	IA1	AH in preoperative biopsy
RS	70	38	IA2	Stage 4 chronic kidney disease on dialysis
RS	73	31	IA1	Voluminous laparocele
RS	77	41	IA2	AH in preoperative biopsy
RS	90	31	IAm(POLE mut)	Dementia
RS	53	35	AH	
RS	84	31	AH	
RS	69	43	IB	Voluminous laparocele
RS	61	40	IIB	BMI
RS	64	41	IA2	Synchronous lung cancer
RS	80	36.5	IA2	AH in preoperative biopsy
RS	67	33	IAm(POLE mut)	Voluminous laparocele
RS	68	36	IICm(p53 mut)	BMI
RS	85	32	IB	Old age
RS	74	50	IA1	AH in preoperative biopsy
RS	86	32	IIA	Old age
RS	58	59	IA2	AH in preoperative biopsy
RS	46	39	AH	
RS	48	67	IA2	BMI
RS	69	28	IA2	AH in preoperative biopsy
RS	59	51	AH	

LS: laparoscopic surgery; RS: robotic surgery; BMI: body mass index; and AH: atypical hyperplasia.

## Data Availability

The data that support the findings of this study are available from the corresponding author, S.R., upon reasonable request.
